# Deconvoluting the dual hypoglycemic effect of wedelolactone isolated from *Wedelia calendulacea*: investigation *via* experimental validation and molecular docking†

**DOI:** 10.1039/c7ra12568b

**Published:** 2018-05-18

**Authors:** Vikas Kumar, Kalicharan Sharma, Bahar Ahmed, F. A. Al-Abbasi, Firoz Anwar, Amita Verma

**Affiliations:** Natural Product Drug Discovery Laboratory, Department of Pharmaceutical Sciences, Faculty of Health Sciences, Sam Higginbottom Institute of Agriculture, Technology & Sciences Allahabad Uttar Pradesh India – 211007; Department of Pharmaceutical Chemistry, SPER, Jamia Hamdard New Delhi-110062 India; Department of Biochemistry, King Abdulaziz University Jeddah-21589 Kingdom of Saudi Arabia; Bioorganic & Medicinal Chemistry Research Laboratory, Department of Pharmaceutical Sciences, Faculty of Health Sciences, Sam Higginbottom University of Agriculture, Technology & Sciences Allahabad-211007 Uttar Pradesh India amitaverma.dr@gmail.com amita.verma@shiats.edu.in

## Abstract

*Wedelia calendulacea* has a long history of use in the Indian Ayurvedic System of Medicine for the treatment, prevention, and cure of a diverse range of human diseases such as diabetes obesity, and other metabolic diseases. A wide range of chemical constituents, such as triterpenoid saponin, kauren diterpene, and coumestans, has been isolated from the plant. Conversely, no published literature is available in relation to the isolation of wedelolactone (WEL) for its anti-diabetic effect. The aim of the present study was to isolate the bioactive phyto-constituent from *Wedelia calendulacea* and to scrutinize the antidiabetic effect with its possible mechanism of action. The structure of the isolated compound was elucidated by different spectroscopy techniques. Proteins, such as dipeptidyl peptidase-4 (DPPIV), glucose transporter 1 (GLUT1), and peroxisome proliferator-activated receptors-γ (PPARγ), were also subjected to *in silico* docking. Later, this isolated compound was scrutinized against α-glucosidase and α-amylase enzyme activity along with an oral glucose tolerance test (OGTT) for estimation of glucose utilization. Streptozotocin (STZ) was used for the induction of type II diabetes mellitus (DM) in Wistar rats. The rats were divided into different groups and received the WEL (5, 10, and 20 mg kg^−1^, b.w.) and glibenclamide (2.5 mg kg^−1^, b.w.) for 28 days. The blood glucose level (BGL), plasma insulin, and body weight were determined at regular time intervals. The serum lipid profile hypolipidemic effect for the different antioxidant markers and hepatic tissue markers were scrutinized along with an inflammatory mediator to deduce the possible mechanism. With the help of spectroscopy techniques, the isolated compound was identified as wedelolactone. In the docking study, WEL showed docking scores of −6.17, −9.43, and −7.66 against DPP4, GLUTI, and PRARY, respectively. WEL showed the inhibition of α-glucosidase (80.65%) and α-amylase (93.83%) and suggested an effect on postprandial hyperglycemia. In the OGTT, WEL significantly (*P* < 0.001) downregulated the BGL, a marker for better utilization of drugs. In the diabetes model, WEL reduced the BGL and enhanced the plasma insulin and body weight. It also significantly (*P* < 0.001) modulated the lipid profile; this suggested an anti-hyperlipidemia effect. WEL significantly (*P* < 0.001) distorted the hepatic tissue, acting as an antioxidant marker in a dose-dependent manner. WEL significantly (*P* < 0.001) downregulated the C-reactive protein (CRP), tumor necrosis factor alpha (TNF-α), and interleukin 6 (IL-6) level. On the basis of the available results, we can conclude that WEL can be an alternative drug for the treatment of type II DM either by inhibiting the production of inflammatory mediator or by the downregulation of oxidative stress.

## Abstract 2

*Wedelia calendulacea* has a long history of use in the Indian Ayurvedic System of Medicine for the treatment, prevention, and cure of a diverse range of human diseases such as diabetes obesity, and other metabolic diseases.

## Graphical abstract

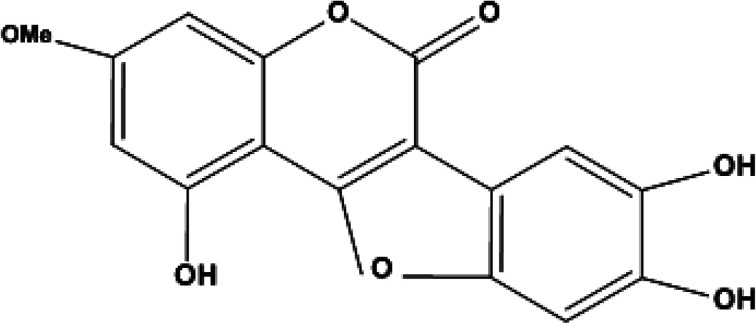

## Introduction

Diabetes mellitus (DM) is a metabolic disease exemplified through the upregulation of BGL causing either an expansion of insulin deficiency or insulin resistance. DM is connected with the development of macrovascular complications, which can further turn and progress into cardiovascular disorders. Nowadays, due to changes in lifestyles, increased obesity, and an aging population, the frequency and occurrence of diabetes is rising.^1,2^ According to the World Health Organization (WHO), millions of people suffer from diabetes, with the number of cases increasing every year, especially in the middle-income countries. Epidemiological studies have suggested that type II diabetes has a higher disease frequency compared to type I diabetes.^3^ Only 10% of patients suffer from type I diabetes, while 90% suffer from type II diabetes among all types of diabetes.^4,5^ A cost evaluation from a recent review study showed that the cost of diabetes treatment is more than the US$ 827 billion, annually. A comparison of the life expectancy for diabetic patients shows it is 10 years less than for non-diabetics. If the pace of this disease maintains the same, it could reach 360–380 million people affected globally by 2025–2030. Therefore, the diabetes disease is a global burden and requires prompt attention. According to the Indian Task Force on Diabetes Care in India, there is a 9% incidence of diabetes in urban areas and 3% in rural areas.^6,7^

Various investigations have suggested that enzymes, namely DPPIV, α-amylase, protein tyrosine phosphatase 1B (PTP1B), and α-glucosidase, can be targeted for the treatment of type II DM. DPPIV is considered as the prime target for glycemic control, and works by inactivation of the glucagon inhibitory peptide, glucagon-like peptide (GLP-1), and incretin hormones. DPP-4 inhibition not only affects the incretins breakdown, but also influences the cytokines and chemokines. A continuous inhibition of DPPIV has a beneficial effect on decreasing HbA1c and postprandial glucose levels.^8,9^ The prolonged inhibition of DPPIV enzymes suggests a novel mechanism to treat type II DM. Such an inhibition is a target for these enzymes for the activation of glucose transport GLUT4 in cell plasma membranes, activation of AMP kinase, and upregulation of the glucose-6P and glycogen stores. Moreover, researchers have diverted their research toward the inhibition of PTP1B, which circulate the insulin signaling *via* dephosphorylating the phosphotyrosine residue on insulin receptors.^8,10^

Nowadays, synthetic drugs and insulin therapy are used for the treatment of type II DM. Conversely, these types of treatment have their own side effects, such as weight gain, dropsy, and drug resistance. Due to the limitation of available drugs for the treatment of DM and the limitations in accessing public health systems in low-income communities, patients are attracted to alternative therapies with fewer side complications. According to reports in the ancient literature, more than 800 medicinal plants have been reported to have antidiabetic activity, while ethnopharmacological survey reports show that more than 1200 medicinal plants are used for diabetes mellitus.^11,12^ Presently, alternative systems of therapies are gaining more popularity due to their high margin of safety profiles. A lot of medicinal plants are available for the treatment of DM. A few of these plants have already been systematically and scientifically screened for antidiabetic potential.

There exist 65 species of *Wedelia* distributed in warm and tropical temperate regions, such as India, China, Ceylon, Japan, and Burma. *Wedelia calendulacea* (Compositae) is a perennial herb commonly cultivated in marshy and wet places in Uttar Pradesh, Arunachal Pradesh, and Assam in India and is also found in the coastal areas of Japan and the rest of the World.^13^ Traditionally, the plant is used as a deobstruent, cholagogue, and in the treatment of hepatics and its disorders. It's also used in the Ayurvedic system of medicine as a Hepatoprotective drug. The Vaidyas (Ayurvedic physician in Tamilnadu) and other traditional physicians use this plant in the treatment of various liver disorders in the form of a liver tonic.^13,14^ The leaves are used in the treatment of cephalalgia, cough, and skin disease in the form of a cream and tonic. Further, it is also used for its effect on swelling, the central nervous system, coughs, baldness, skin disease, and diarrhea.^15,16^

Norwedelolactone and norwedelic acid have also been isolated from the plant,^17^ while three bisdesmosidic oleanolic acid saponins have been isolated from the plant (fresh plant), one of which is a new bisdesmosidic oleanolic acid saponin. The second saponin has been identified as a known ginsenoside. The structure of the third saponin has not been fully worked out but it appears to be a bisdesmosidic oleanolic acid glycoside with a β-d-glucosidal moiety at position-28 of oleanolic acid.

No detailed anti-diabetic effect of isolated wedelolactone from *Wedelia calendulacea* had been previously scrutinized against STZ-induced type II DM. Therefore, in the current study, we investigated the anti-diabetic effect of isolated wedelolactone to find the possible mechanism of action. In the current study, we hypothesized that wedelolactone produced its anti-diabetic effect to either inhibit the production of inflammatory mediator or by downregulation of oxidative stress.

## Material and methods

### Plant material

The plant material *Wedelia calendulacea* was collected from the market and authenticated by Dr M. P. Sharma, Department of Botany, Jamia Hamdard, and the prepared herbarium was submitted to the department for further reference.

### Extraction and isolation

The collected *Wedelia calendulacea* plant material was dried in the shade and crushed to produce a coarse powder. The obtained powder was subjected to methanol for further extraction. The ethanolic extract was further fractionated with petroleum ether, chloroform, and methanol.^18^

The methanolic extract was used for column chromatography in a silica gel (60–120 mesh) slurry and the column was packed with petroleum ether. The packed column was eluted *via* using different polarity solvents (pet ether, chloroform, and methanol).

### Dry lab study (*in silico* docking)

A molecular docking study was carried out using the Schrodinger software suite (Maestro, version 9.4). The build panel was used to prepare the 3D sketched format of the ligand and docking was used for preparing the ligrep application. For the molecular docking study, the protein (PDB ID: 2G63, 5EQG and 2PRG) was selected from the protein data bank, followed by removing the solvent and adding the hydrogen, for minimization of the presence of the standard ligand *via* the preparation of a protein wizard. For validation of the docking study, the standard (glibenclamide) was re-docked to calculate the RMSD.

### *In vitro* α-glucosidase activity

The inhibitory α-glucosidase effect of WEL was scrutinized by using the reported model of Ahmed *et al.* (2014) and Kumar *et al.* (2015)^19,20^ with minor modification. A 96-well microtiter plate was used for estimation of the inhibition of α-glucosidase enzymes. The different concentrations of WEL were mixed with the enzyme (20 μl) solution, containing 0.8 U ml^−1^ of α-glucosidase in 0.01 M PBS, and then incubated at room temperature for 15 min. To terminate the reaction, 0.2 M Na_2_CO_3_ was mixed in PBS (0.1 M) and the absorbance of the mixture was estimated at 405 nm. The results are presented herein as a percentage of the inhibition of α-glucosidase and α-amylase *via* using the following formula:
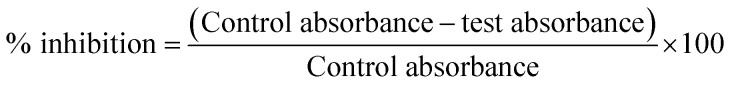


### α-Amylase activity

In order to scrutinize the effect of WEL, α-amylase enzymes activity was assessed using the reported model of Ali *et al.* (2006)^21^ with minor modification. Briefly, the test sample (WEL and acarbose) was mixed and pre-incubated with sodium phosphate (20 mM; pH = 6.7) for 5 min and the final volume was made up to 2 ml *via* adding the starch (2% w/v) and the mixture was then incubated again for 5 min at room temperature. After the incubation period, di-nitro salicylic acid (1 ml) was added. The reaction mixture was kept in a boiling water bath for 5 min. After that, the reaction mixture was cooled down in an ice bath and again deionized water was added and the absorbance of the reaction mixture was estimated at 540 nm. The percentage of inhibition was determined *via* using the pre-determined formula.

### Animals

Wistar rats (150–180 g, body weight; same age) were used for the current experimentation study. The rats were kept in single polyethylene cages and retained under standard laboratory conditions (40–60% humidity; 22 ± 5 °C and 12/12 h day/night cycle). The rats received a standard diet (food chew), purchased from Hindustan Liver Limited, Mumbai, India, and received water *ad libitum*. The current research protocol was approved by the Institutional Animal Ethical Committee (IAEC) as per the instruction for the Purpose of Control and Supervision of Experiments on Animals (CPCSEA) (IAEC/SHUATS/PA/2017XII/SVS03).

### Oral glucose tolerance test (OGTT test)

Kumar *et al.* and Ahmed *et al.*'s methods was used for the oral glucose tolerance test with minor modification.^19,22^ Swiss albino Wistar rats were grouped in seven groups:

Group I: normal and treated with saline only,

Group II: normal and treated with WEL (20 mg kg^−1^),

Group III: glucose and treated with saline only (1 ml kg^−1^),

Group IV: glucose and treated with WEL (5 mg kg^−1^),

Group V: glucose and treated with WEL (10 mg kg^−1^),

Group VI: glucose and treated with WEL (20 mg kg^−1^),

Group VII: glucose and treated with glibenclamide (2.5 mg kg^−1^).

The blood glucose levels of all the groups rats were determined at 0 h and after that, all the groups rats received glucose (2 mg kg^−1^), except NC and NC received WEL (20 mg kg^−1^). Blood samples of all the groups rats were withdrawn at regular intervals (0, 30 60, 90, and 120 min) to determine the blood glucose level (BGL).

### Experimental protocol

The rats were grouped as following, with each group containing 6 rats:

Group I: normal and treated with saline only (1 ml kg^−1^ per day) for 28 days,

Group II: normal and treated with WEL (20 mg kg^−1^ per day) for 28 days,

Group III: diabetic and treated with saline only (1 ml kg^−1^ per day) for 28 days,

Group IV–VI: diabetic rats treated with WEL (5, 10, and 20 mg kg^−1^ per day) for 28 days,

Group VII: diabetic and treated with glibenclamide (2.5 mg kg^−1^ per day) for 28 days.

The rats received the above-discussed treatment for 28 days. The body weight and food intake were monitored at regular intervals. The BGL and plasma insulin levels were also determined at a regular interval.^23^

### Ponderal homogeneity index (iPH) and ponderal grain (PG)

The ponderal homogeneity index and the ponderal gain were estimated by using the formula:
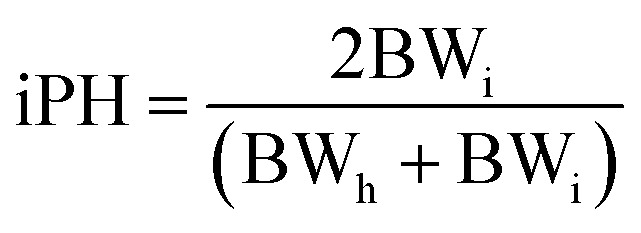
where BW_i_ = initial body weight, BW_h_ = highest body weight.
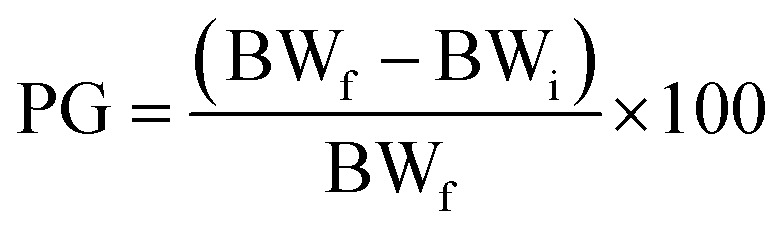
where BW_i_ = body weight (initial), BW_f_ = body weight (final).

### Biochemical parameters

Blood samples were collected by retro-orbital plexus puncture, and then centrifuged (5000 rpm) for 15 min to collect the serum for estimation of blood glucose, plasma level, and other parameters. The hepatic parameters, namely alkaline phosphatase (AST), aspartate aminotransferase (AST), alanine transaminase (ALT); serum renal parameters, including blood urea nitrogen (BUN), total protein, creatinine, albumin; lipid parameters, such as total cholesterol (TC), high density lipoprotein (HDL), triglyceride (TG), very low density lipoprotein (VLDL) and, low density lipoprotein (LDL), were scrutinized using commercially available diagnostic kits. The atherogenic index and coronary risk index were scrutinized as per the given formula:
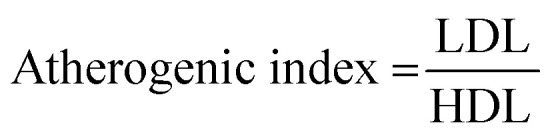

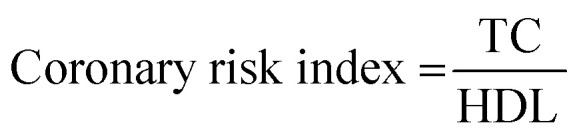


### Antioxidant parameters

The antioxidant parameters, such as malonaldehyde (MDA), glutathione peroxidase (GPx), superoxide dismutase (SOD) and catalase (CAT), were determined by the reported method of Anwar *et al.*, Afzal *et al.*, Khan *et al.*, Kumar *et al.*, and Verma *et al.*, with minor modification.^24–31^

### Inflammatory and pro-inflammatory mediators

Pro-inflammatory cytokines, namely interleukin-6 (IL-6), tumor necrosis factor-α (TNF-α), and inflammatory mediators, such as C-reactive protein (CRP), were also estimated as per the instructions in the available commercial kits.

### Statistical analysis

In the experimental study, all the data are reported with the mean SEM and analysis of variance (ANOVA) *via* using the Graph Pad Prism software version 5.0 (U. S. A), where *P* values such as *p* < 0.05, *p* < 0.01, and *p* < 0.001 are considered as significant.

## Results

### Characterization of compound WC-1

**Fig. 1:**
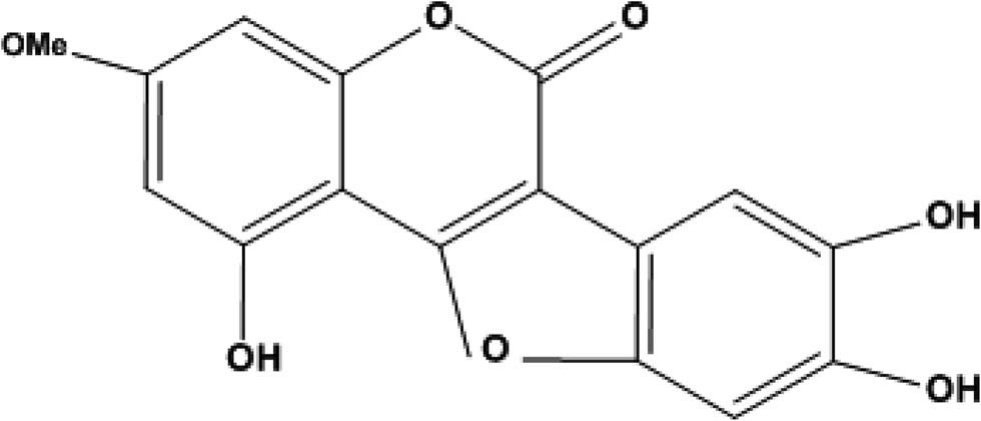
Structure of wedelolactone.

**Fig. 2:**
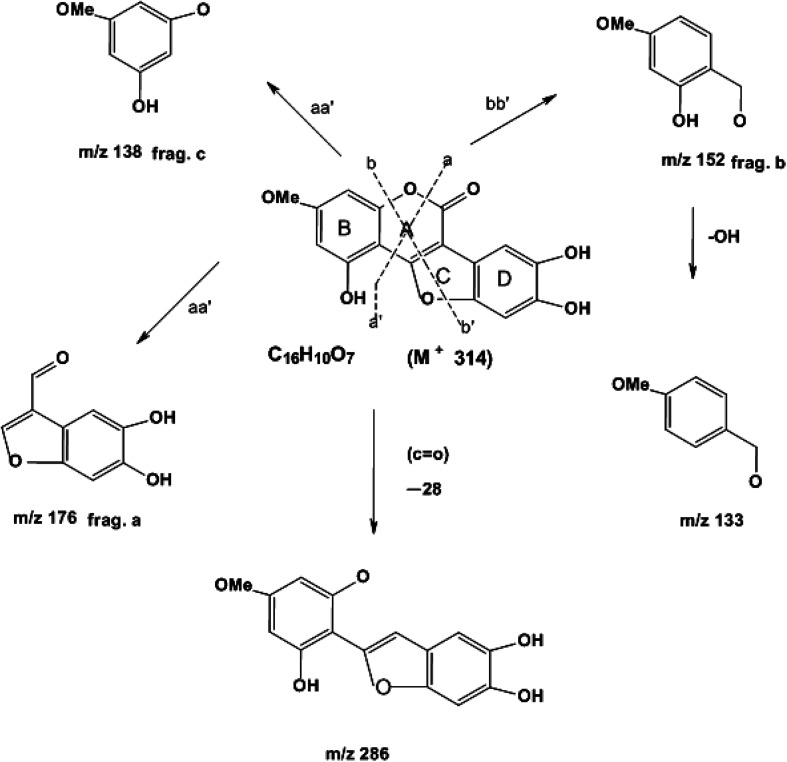
Mass fragmentation of wedelolactone.

Elution of the column with CHCl_3_–MeOH (95 : 5) yielded WEL (Fig. 1) as a light green amorphous powder, 95 mg (Fig. 2).

*R*_f_: 0.58 (CHCl_3_–MeOH: 9 : 1).

M.p.: >300 °C.

UV: *λ*_max_ 352, 304, 294, 249 nm (ESI Fig. 1†).

IR (KBr): *ν*_max_ 3305 (OH), 1717 (δ-lactone carbonyl), 1619 (C=C), 1200 (C–O, furan), 1060 (C–O, phenolic), 1448, 1319, 1149, 953, 863 cm^−1^ (ESI Fig. 2†).

1D NMR (DMSO): shown in Table 1 (ESI Fig. 3, 4 and Table 1† shows the NMR data).

**Table 1:** 1D NMR spectral data of compound WC-1

Position	^1^H NMR	^13^C NMR
1	—	—
2	—	161
3	—	154
4	—	100.8
5	—	153
6	6.38 (d, *J* = 2.0)	97.1
7	—	158
8	6.38 (d, *J* = 2.0)	97.6
9	—	95.8
10	7.08 s	91.9
11	—	143.8
12	—	142.7
13	7.26 s	103
14	—	157
15	3.73 s	54.2
—	8.98 brs at 11,12 (OH)	—
—	10.58 s at 5 (OH)	—

EIMS (probe) 70 eV, *m*/*z* % (rel. int): 314 (M)^+^ (C_16_H_10_O_7_) (100), 286 (15), 176 (70), 152 (20), 138 (58) (ESI Fig. 5†).

### Dry lab activity (*in silico* docking)

**Fig. 3:**
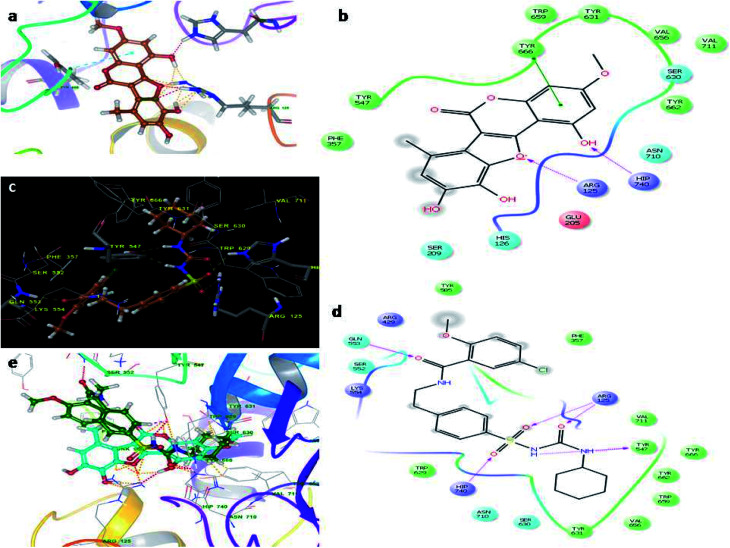
Molecular docking studies of wedelolactone and glibenclamide against DPP4 catalytic site (PDBID-2G63). (a) Binding interaction of WEL, (b) Ligplot of WEL against DPP4 catalytic site, (c) binding interaction of glibenclamide against DPP4 catalytic site, (d) Ligplot of glibenclamide against DPP4 and (e) superimposition of WEL with glibenclamide against DPP4 catalytic domain.

Molecular docking studies were carried by the automated docking protocol. Fig. 3 shows the binding poses of the WEL and glibenclamide. As can be observed, the docking program positioned compounds WEL to make hydrogen bonds with His740 and arg125 and also makes pi–pi stacking with tyr666 against a backbone of DPP4 protein. This gives a better interaction with the backbone of the DPP4 protein due to its hydrophilic interaction and pi–pi interaction with the amino acid residue of DPP4 protein. We further validated the interaction *via* superimposing our ligand WEL with glibenclamide and observed that the binding orientation of our ligand was almost similar to WEL (Table 2).

**Table 2:** Docking scores of wedelolactone and glibenclamide against DPP4, GLUT1, and PPARY protein

S. no	Ligands	Docking score		
		DPP4	GLUT1	PPARY
1	Wedelolactone	−6.23	−8.98	−7.98
2	Glibenclamide	−6.05	−8.29	−6.73

**Fig. 4:**
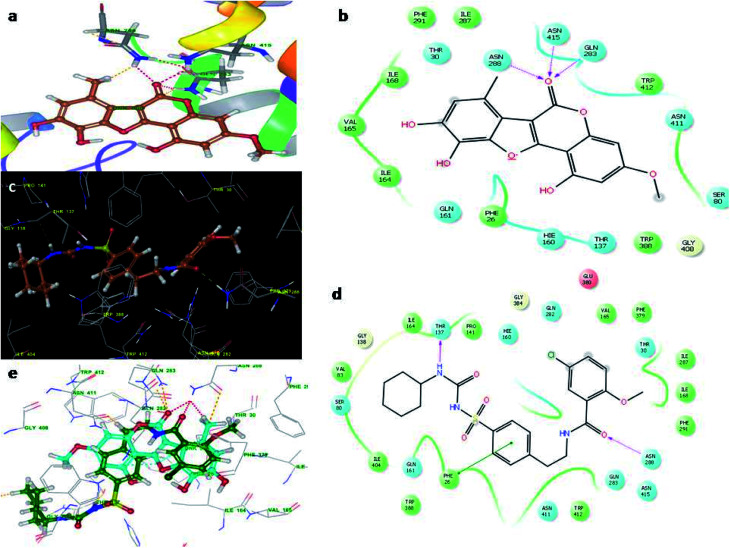
Molecular docking studies of wedelolactone and glibenclamide against GLUT1 catalytic site (PDBID-5EQG). (a) Binding interaction of WEL, (b) Ligplot WELagainst GLUT1 catalytic site, (c) Binding interaction of glibenclamide against GLUT1 catalytic site, (d) Ligplot of glibenclamide against GLUT1 and (e) superimposition of WEL with glibenclamide against GLUT1 catalytic domain.

**Fig. 5:**
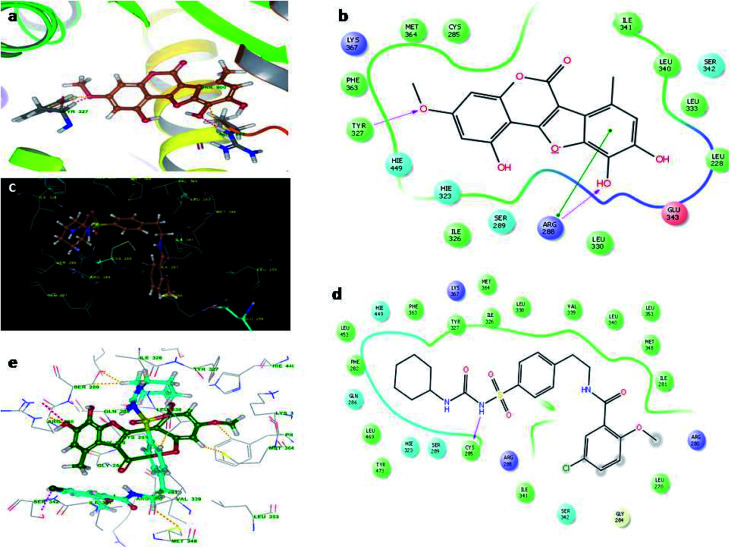
Molecular docking studies of wedelolactone and glibenclamide PPARY catalytic site (PDBID-2PRG). (a) Binding interaction of WEL, (b) WEL against PPARY catalytic site, (c) binding interaction of glibenclamide against PPARY catalytic site, (d) Ligplot of glibenclamide against PPARY and (e) superimposition of WEL with glibenclamide against PPARY catalytic domain.

Fig. 4 illustrates the binding pose of the WEL and glibenclamide ligands. The docking program positioned compounds WEL to make hydrogen bonds with Gln283, Asn288, and Asn415 against the backbone of the GLUT1 protein. Here, a better interaction with the backbone of GLUT1 protein was observed due hydrophilic interactions and pi–pi interactions with the amino acid residue of the GLUT1 protein. We further validated the interaction by superimposing our ligand (WEL) with the glibenclamide (standard) ligand and observed that the binding orientation of our ligand was similar to that of WEL. Fig. 5 reveals the binding poses of the WEL and glibenclamide ligands. Fig. 5 also shows the docking positioned compounds where WEL makes hydrogen bonds with arg288 and tyr327 and also gives pi–pi stacking with Arg288 against the backbone of the PPARY protein. Herein, WEL gives better interaction with the backbone of PPARY protein because it exhibits a hydrophilic interaction and pi–pi interaction with the amino acid residue of the PPARY protein. We further validated the interaction by superimposing our ligand (WEL) with the glibenclamide (standard) ligand and observed that the binding orientation of our ligand was similar to the WEL.

### Effect of WEL on α-glucosidase and α-amylase activity

Table 3 shows the effect of WEL on the α-glucosidase and α-amylase enzyme activity. WEL exhibited an inhibition activity on α-glucosidase (80.65 ± 2.13) and α-amylase (93.83 ± 1.82) enzymes. Acarbose also showed the inhibition of α-glucosidase (80.65 ± 2.13) and α-amylase (42.23 ± 1.02) enzymes.

**Table 3:** Effect of WEL on the α-glucosidase and α-amylase inhibition activity

S. no	Sample	% inhibition	
		α-Glucosidase	α-Amylase
1	Wedelolactone	80.65 ± 2.13	93.83 ± 1.82
2	Acarbose	48.92 ± 1.23	42.23 ± 1.02

### Effect of WEL on the oral glucose tolerance test (OGTT)

**Fig. 6:**
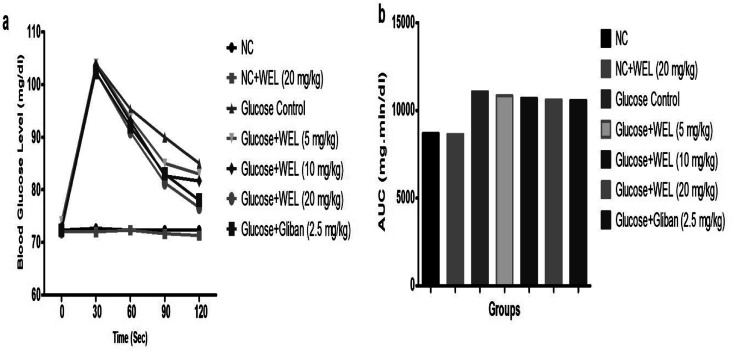
Effect of wedelolactone and glibenclamide on the oral glucose tolerance test (OGTT). (a) Blood glucose level at different time intervals and (b) area under curve (AUC) on the different groups of rats.

The OGTT was executed for the estimation of postprandial hyperglycemia. During the OGTT experimental study, the blood glucose of NC and the NC received WEL (20 mg kg^−1^) exhibited an almost constant BGL at the end of the experimental study. The glucose control group rats showed an upregulation of BGL at the end of the study (Fig. 6). The BGL of the glucose control group rats increased till the 90 min after the glucose administration and then slightly decreased in the last 30 min. But WEL treatment showed a reduced BGL from the starting time and this continued to decrease the BGL till the end of the experimental study in a concentration-dependent manner. The result suggests the WEL-treated rats had better glucose utilization.

### Effect of WEL on body weight

**Fig. 7:**
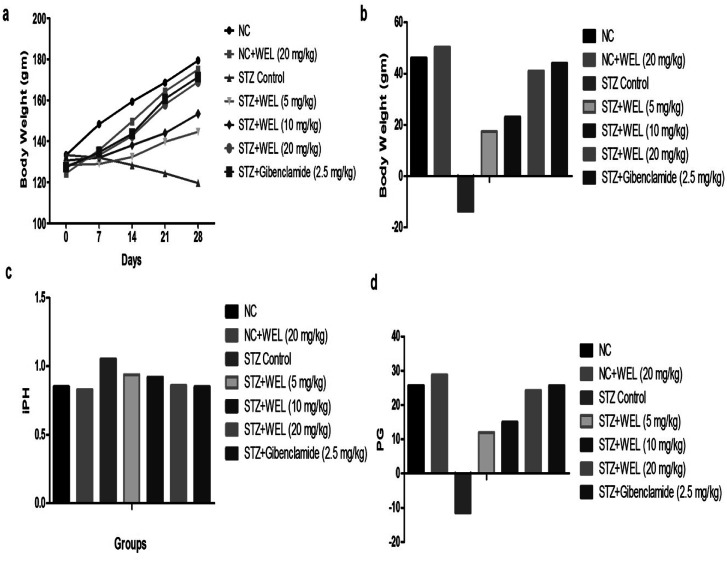
Effect of wedelolactone and glibenclamide on the body weight effect of the streptozotocin (STZ)-induced type II diabetic mellitus rats. (a) Body weight at different time intervals, (b) body weight variation, (c) ponderal homogeneity index (iPH) and (d) ponderal grain (PG).

The NC and NC treated WEL group rats demonstrated an augmented body weight as compared to STZ-induced type II DM rats. On the contrary, the STZ-treated rats treated with WEL illustrated a significantly (*p* < 0.001) augmented body weight in a concentration-dependent manner (Fig. 7a). The NC group rats showed an increase of 1.64 g gain body weight per day throughout the experimental study. NC group rats treated with WEL (20 mg kg^−1^) revealed a 1.78 g gain in body weight per day till the end of the experimental study. STZ-induced type II DM rats showed a 0.49 g reduced body weight per day. STZ-induced rats treated with WEL showed a gain in body weight of 0.62, 082, and 1.46 g per day at doses of 5, 10, and 20 mg kg^−1^. Glibenclamide-treated drugs showed a growth in gain rate of 1.56 g per day as compared to the initial body weight (Fig. 7b).

The ponderal homogeneity index (iPH) of NC and NC receiving WEL (20 mg kg^−1^) showed values of 0.85 and 0.83, respectively, which were considerably increased in the STZ-induced type II DM group rats. WEL treatment to STZ-induced type II DM rats showed a downregulation of the iPH level at a dose-dependent manner, and WEL (20 mg kg^−1^) showed an iPH almost near to the NC group (Fig. 7c). The ponderal grain (PG) was found to be almost equal in the NC and NC rats treated with WEL (20 mg kg^−1^), while the STZ-induced type II DM group exhibited the opposite trend of PG, which was decreased to −11.53 g in a dose-dependent treatment manner with WEL for the upregulation of PG. The same result was found in the glibenclamide-treated group rats (Fig. 7d).

### Effect of WEL on blood glucose and insulin level

**Fig. 8:**
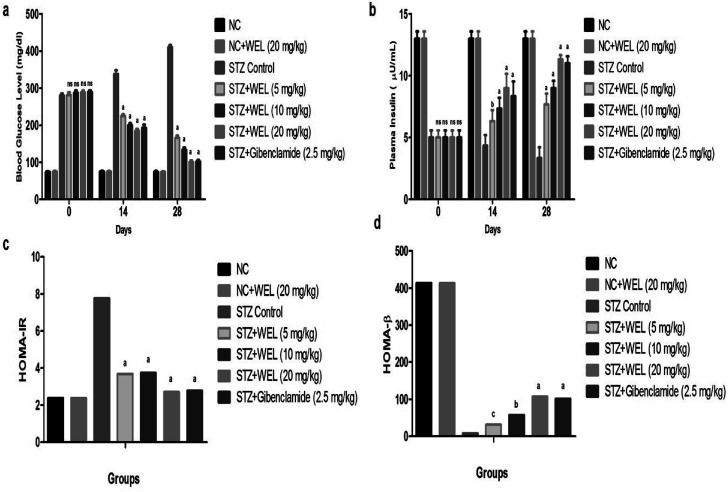
Effect of wedelolactone and glibenclamide on the blood glucose level of the streptozotocin (STZ)-induced type II diabetic mellitus rats. (a) Blood glucose level at different time intervals, (b) plasma insulin level, (c) HOMA-IR and (d) HOMA-β.

The BGL of the NC and NC received treated group rats showed initial BGLs of 74 ± 1.32 and 74.67 ± 1.89; and were almost the same at 74.33 ± 1.65 and 74.23 ± 1.59 at the end of the experimental study. The initial BGL of the STZ-induced type II DM group rats was 280 ± 4.83, which was significant at 410.64 ± 5.34 till the end of the study. The STZ-induced type II DM group rats treated with WEL (5, 10, and 20 mg kg^−1^) showed BGLs of 281.67 ± 3.45, 287.21 ± 4.93, and 288 ± 3.84 mg dl^−1^ at day 0, which were significantly (*P* < 0.001) reduced to 165.67 ± 2.57, 134 ± 3.42, and 100 ± 2.87 mg dl^−1^ at day 28, respectively. These results suggest the antidiabetic effect of WEL *via* reducing the BGL at the end of the experimental study (Fig. 8a).

The plasma insulin level of the NC and NC received WEL (20 mg kg^−1^) groups had almost similar levels at 13 ± 0.98 and 13 ± 0.84 at day 0 and 13 ± 0.67 and 13 ± 0.49 at day 28. STZ-induced type II DM group rats showed the decreased plasma insulin level of 5 ± 0.64 (μU ml^−1^) due to toxicity induced in the pancreas tissue. WEL (5, 10, and 20 mg kg^−1^)-treated group rats showed plasma insulin levels of 5.03 ± 0.73, 5.08 ± 0.62 and 5.09 ± 0.93 (μU ml^−1^) at day 0, which were significantly (*P* < 0.001) increased to 7.65 ± 0.94, 9.01 ± 0.73, and 11.45 ± 0.83 (μU ml^−1^) at day 28 in a dose-dependent manner. On the other hand, the STZ-induced type II DM group rats treated with glibenclamide showed a significant (*P* < 0.001) increase in plasma insulin level from 5.06 ± 0.43 to 11 ± 0.83 (μU ml^−1^), which suggested an antidiabetic effect. This effect occurred due to the improved dysfunction of pancreatic β-cells (Fig. 8b).

HOMA-IR was increased and HOMA-β was decreased in the STZ-induced type II DM group rats as compared to NC and NC received WEL (20 mg kg^−1^) group rats. WEL treatment showed a reduced level of HOMA-IR (Fig. 8c) and enhanced level of HOMA-β (Fig. 8d) as compared to the STZ-induced type II DM group rats, which suggested an improvement of the insulin level and an antidiabetic effect.

### Effect of WEL on antioxidant parameters

**Fig. 9:**
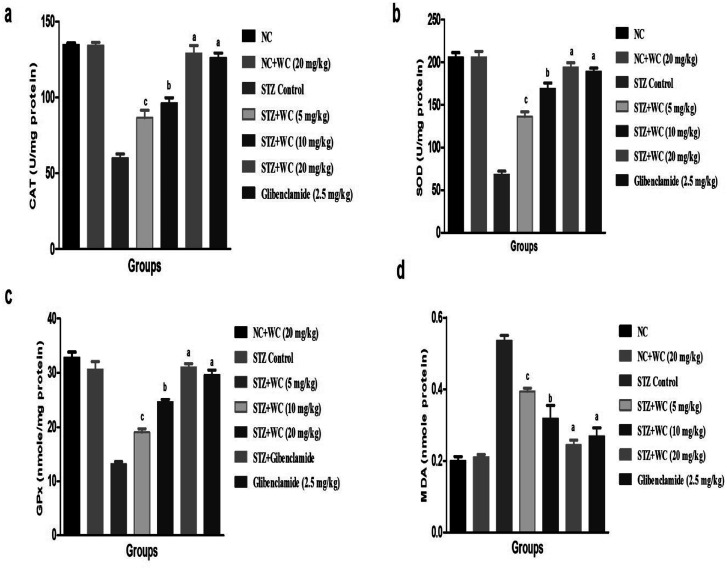
Effect of wedelolactone and glibenclamide on the antioxidant parameters of the streptozotocin (STZ)-induced type II diabetic mellitus rats. (a) CAT, (b) SOD, (c) GPx and (d) MDA. The comparisons were made by ANOVA followed by Dunnett's test. ^c^*P* < 0.05 is considered significant, ^b^*P* < 0.01 is considered very significant, ^a^*P* < 0.001 is considered extremely significant.

Fig. 6 clearly shows the effect of WEL on the STZ-induced type II DM group rats. NC (134.6 ± 4.32 U mg^−1^ protein) and NC (134 ± 5.19 U mg^−1^ protein) group rats treated with WEL exhibited similar levels of antioxidant markers. STZ-induced type II DM rats showed a reduction in the level of CAT (59.8 ± 3.87 U mg^−1^ protein) in comparison with the NC group rats. WEL (5, 10, and 20 mg kg^−1^) treatment showed a significantly (*P* < 0.001) increased (86.6 ± 3.93, 96.2 ± 3.23, and 129 ± 4.92 U mg^−1^ protein) level of CAT as compared to the STZ-induced type II DM group rats (Fig. 9a).

A similar trend was observed in the SOD (68 ± 2.46 U mg^−1^ protein) (Fig. 9b) and GPx (13.2 ± 1.06 U mg^−1^ protein) antioxidant marker in the STZ-induced type II DM group rats, while WEL treatment showed the upregulation of SOD (193.8 ± 4.91 U mg^−1^ protein) and GPx (31.02 ± 1.16 U mg^−1^ protein) antioxidant marker levels as compared to the STZ-induced type II DM group. A same improvement of the SOD (188.6 ± 5.12 U mg^−1^ protein) and GPx (29.6 ± 1.82 U mg^−1^ protein) antioxidant marker was observed in the glibenclamide group rats (Fig. 9c).

In terms of the MDA level, STZ-induced type II DM group rats showed an increased level of MDA (0.536 ± 0.08 nmole protein), which was significantly (*P* < 0.001) reduced by WEL treatment (0.244 ± 0.02 nmole protein). Glibenclamide-treated rats showed an increased MDA level (Fig. 9d).

### Effect of WEL on hepatic enzyme parameters

**Fig. 10:**
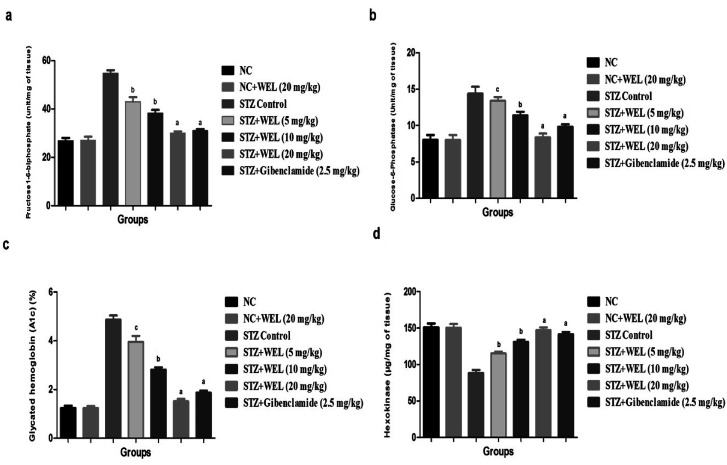
Effect of wedelolactone and glibenclamide on the hepatic parameters of the streptozotocin (STZ)-induced type II diabetic mellitus rats. (a) fructose-1-6-biphosphate, (b) glucose-6-phosphatase, (c) glycalated hemoglobin and (d) hexokinase. The comparisons were made by ANOVA followed by Dunnett's test. ^c^*P* < 0.05 is considered significant, ^b^*P* < 0.01 is considered very significant, ^a^*P* < 0.001 is considered extremely significant.

Fig. 10 illustrates the effect of WEL on the normal and STZ-induced type II DM group rats. STZ-induced type II DM group rats showed the upregulation of fructose1-6-biphosphate, glucose-6-phosphatase, and glycated hemoglobin, and downregulation of the hexokinase level as compared to NC and NC rats treated with WEL (20 mg kg^−1^) (Fig. 7). WEL treatment significantly (*P* < 0.001) altered the level of diabetic parameters in a dose-dependent manner. Glibenclamide showed a modulation of the level of the diabetic parameters in comparison with the STZ-induced type II DM group rats.

### Effect of WEL on lipid profile

**Fig. 11:**
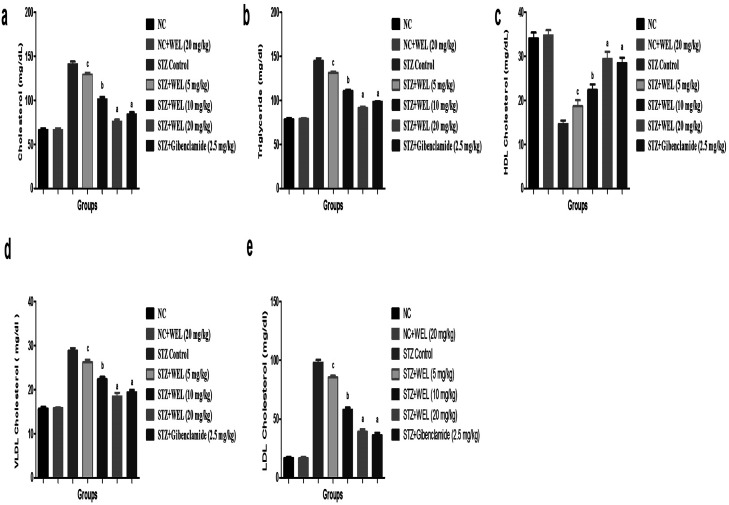
Effect of wedelolactone and glibenclamide on the lipid parameters of the streptozotocin (STZ)-induced type II diabetic mellitus rats. (a) total cholesterol, (b) triglyceride, (c) HDL cholesterol, (d) VLDL cholesterol and (e) LDL cholesterol. The comparisons were made by ANOVA followed by Dunnett's test. ^c^*P* < 0.05 is considered significant, ^b^*P* < 0.01 is considered very significant, ^a^*P* < 0.001 is considered extremely significant.

Fig. 11 reveals the effect on the normal control and WEL on the lipid profile. Alteration of the lipid profile is a crucial parameter, which occurs due to diabetes. STZ-induced type II DM group rats showed an increased level of triglycerides, total cholesterol, LDL, and VLDL, and reduced HDL level at the end of the experimental study as compared to the normal control and normal control treated with WEL (20 mg kg^−1^) (Fig. 11). STZ-induced type II DM group rats treated with WEL showed an alteration of the lipid profile and showed the effect of WEL against lipid abnormality.

### Effect of WEL on the coronary risk index and atherogenic index

**Fig. 12:**
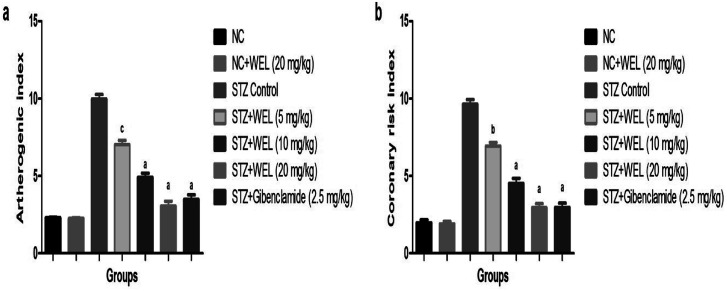
Effect of wedelolactone and glibenclamide on the atherogenic and coronary risk index of the streptozotocin (STZ)-induced type II diabetic mellitus rats. (a) atherogenic index and (b) coronary risk index. The comparisons were made by ANOVA followed by Dunnett's test. ^c^*P* < 0.05 is considered significant, ^b^*P* < 0.01 is considered very significant, ^a^*P* < 0.001 is considered extremely significant.

Coronary risk factor and an atherogenic index were amplified in the STZ-induced type II DM rats, while the dose-dependent treatment of WEL showed a reduction in the level of coronary risk factor and the atherogenic index in a concentration-dependent manner and suggested a cardio-protective effect of WEL (Fig. 12).

### Effect of WEL on inflammatory markers

**Fig. 13:**
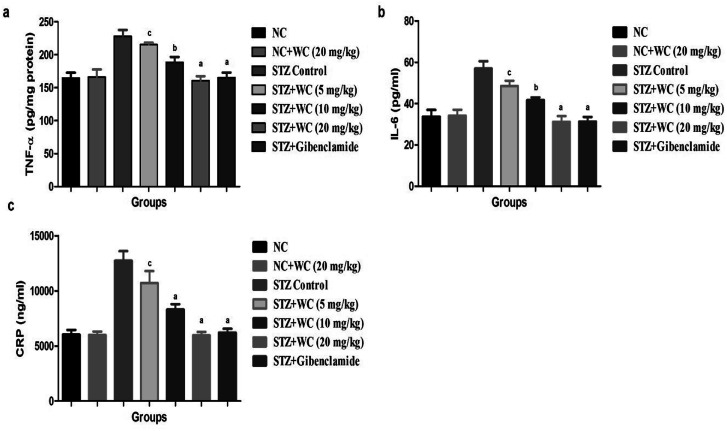
Effect of wedelolactone and glibenclamide on the inflammatory mediators of the streptozotocin (STZ)-induced type II diabetic mellitus rats. (a) TNF-α, (b) IL-6 and (c) CRP. The comparisons were made by ANOVA followed by Dunnett's test. ^c^*P* < 0.05 is considered significant, ^b^*P* < 0.01 is considered very significant, ^a^*P* < 0.001 is considered extremely significant.

Inflammatory markers, such as TNF-α, IL-16, and CRP, were estimated in the normal control and WEL-treated group rats. The level of TNF-α was upregulated in the STZ-induced type II DM group as compared to the NC and NC treated rats with WEL (20 mg kg^−1^). A level of TNF-α almost double was found in the STZ-induced type II DM group, which was significantly (*P* < 0.001) downregulated by the WEL in a dose-dependent manner (Fig. 13a).

The same downregulation trend was found in the IL-6 and CRP levels of the STZ-induced type II DM group as compared to the NC and NC treated rats with WEL (20 mg kg^−1^), which was significantly (*P* < 0.001) reduced by the WEL. On the other hand, glibenclamide significantly (*P* < 0.001) reduced the level of the above-discussed inflammatory mediators (Fig. 13b and c).

## Discussion

Compound wedelolactone (WEL) was obtained as a green amorphous powder corresponding to a molecular formula of C_16_H_10_O_7_, as established by different spectroscopy techniques, such as DEPT spectra, mass spectrum (M^+^ 314), and ^13^C NMR chromatographic observation of TLC for the isolated compound suggested a bright blue fluorescence and generated a green color with FeCl_3_. In the UV spectrum, we found a few peaks at 249, 294, 304, and 352 nm. The IR spectrum showed peaks at 3305 cm^−1^, suggesting the presence of a hydroxyl group, δ-lactone carbonyl (1717 cm^−1^), and a double bond (1619 cm^−1^). The ^13^C NMR and ^1^H NMR showed 16 carbon atoms for the molecule, consisting of eleven quaternary, four methane, and one methoxy carbon atoms (in total C_16_H_10_). The ^1^H NMR spectrum illustrated two proton doublets at *δ*_H_ = 6.38 attributable to positions 6 and 8, while other singlets at *δ*_H_ = 7.26 (*δ*_c_ = 91.9) and *δ*_H_ = 7.26 (*δ*_c_ = 103) were also obtained in the spectrum, which could be assigned to positions 10 and 13 of the ring D. The ^13^C NMR spectrum exhibited peaks in the downfield region at *δ*_c_ = 153, 143.8, and 142.7 due to carbons at positions 5, 11, and 12 containing the hydroxyl groups. The ^1^H NMR spectrum also exhibited a broad signal of two protons due to hydroxy groups at positions 11 and 12, and a singlet of one proton at *δ*_H_ = 10.58, which was further downfield due to hydrogen bonds with the oxygen of ring C, and thus was assignable to position 5. The ^1^H NMR spectrum demonstrated a three protons singlet at *δ*_H_ = 3.73 (*δ*_c_ = 54.2) due to a methoxyl group assignable at C-7. Other peaks in the ^13^C NMR and ^1^H NMR also provided support for the proposed structure of wedelolactone.

The mass fragmentation pattern revealed prominent peaks at *m*/*z* 314 due to a molecular ion peak, *m*/*z* 286 due to removal of a carbonyl group of ring A, *m*/*z* 176 (frag. a) and *m*/*z* 138 (frag. c) from the rupture of ring A by aa′, and *m*/*z* 152 (frag. b) by the rupture of ring A and ring C by bb′. Other peaks in the spectrum were also in good agreement with the structure. Thus on the basis of the above peaks, we elucidated the structure of wedelolactone as 5-hydroxy-7-methoxy-11,12-dihydroxy-benzofurano coumarin.

Streptozotocin (STZ), a nitrosourea compound obtained from the soil microbe (*Streptomyces achromogenes*), has been widely exploited for the induction of type I (high dose) and type II (low dose) diabetes mellitus in an experimental model of animals. STZ-induced type I and type II DM due to a specific alkylating potency. Due to the presence of a methyl nitrosourea moiety, it induces cytotoxicity into the pancreatic islet of β-cells in the experimental animals *via* the induction of DNA methylation and resultant damage to the DNA.^1,2,32^ Damaging DNA further activated the poly(ADP-ribose) polymerase, starting the depletion of NAD^+^. It also acts as a nitric acid donor in pancreatic cells, which boosts the production of superoxide radical fragmentation into the islet cells, with the resultant death of pancreatic β-cells *via* necrosis and apoptosis. STZ exerts a diabetogenic effect *via* damaging the pancreatic β-cells, imitating DM.^1,2,32^ In the current experimental study, wedelolactone (coumestan) isolated from the *Wedelia calendulacea*, possessed various types of pharmacological benefits. Various categories of oral synthetic hypoglycemic drugs, including sulphonylureas, α-glucosidase, biguanides, thiazolidinediones, and a lot more are available in the market for the treatment of DM. In the current experimental study, we selected glibenclamide (sulphonylureas class of drug), which has a mechanism on the insulin secretion.

α-Glucosidase and α-amylase (carbohydrate hydrolyzing enzymes) are responsible for postprandial hyperglycemia. Acarbose inhibitor, an inhibitor of α-glucosidase and α-amylase, acts *via* blocking the activity of both enzymes leading to downregulation of the polysaccharides breakdown and thereby downregulating the boosting post-prandial in BGL and additionally inducing weight loss.^33^ α-Amylase initiates the process by the digestion of carbohydrates through the breakdown of 1,4 glycosidic linkages of polysaccharides to disaccharides, which further convert into monosaccharides *via* α-glucosidase enzymes, which leads to postprandial hyperglycemia.^34^ WEL showed an inhibition of enzymatic activity, suggesting the therapeutic targets for type II DM. The synthetic drug showed a higher inhibition of acarbose inhibitor of α-amylase activity; conversely, the use of the synthetic drug is limited due to their specific side effects, namely meteorism, distention, diarrhea, and abdominal distention, which occur due to the excessive reduction of α-amylase. The previous investigations suggested that a strong inhibition of α-glucosidase and mild reduction of α-amylase exhibited by various plant-based compounds could be responsible for there being fewer side effects.^35^ Therefore, the inhibitory effect of α-glucosidase and α-amylase are considered as one of the important mechanism to control hyperglycemia *via* delaying the digestion of carbohydrates, which continues to downregulate postprandial hyperglycemia. The same result was observed by the WEL *via* the inhibition of α-glucosidase and α-amylase activity, which suggests it could be used as an alternative anti-diabetic drug with less side effects.

Insulin resistance plays a significant phase in metabolic syndrome, representing the major risk factor for the expansion of diabetes mellitus and glucose intolerance.^36^ Downregulation of the insulin resistance postpones the augmentation of type II DM and its complications. The OGTT test was performed for scrutinizing the effect of the drug on the utilization of glucose. In the OGTT test, glucose disposal was observed to be considerably upregulated and downregulated for blood glucose, and it was observed for up to 6 h.^2,37^ OGTT control group rats exhibited increased BGL till 6 h. On the contrary, WEL treatment group rats had reduced BGL, suggesting the better utilization of glucose in the treated rats.^1^ Glibenclamide-treated group rats showed similar results, and so on the basis of a comparison of the results, we can conclude that WEL has a similar effect to glibenclamide on the OGTT rats *via* the downregulation of insulin resistance.

STZ-induced DM rats served as hyperglycemia interconnected with the downregulation and discharge of insulin secretion. This occurs due to damage of pancreatic β-cells by STZ-induced toxicity in the pancreas. STZ-induced DM rats showed a boosted level of fasting BGL due to the damage of pancreatic β-cells.^1,32^ The oral administration of WEL and glibenclamide showed an improvement in insulin secretion from existing and regenerated β-cells of the pancreas.

STZ-induced DM rats showed weight loss throughout the experimental study. The reduced body weight was observed in the rats due to amplifying the muscle destruction and degradation of structural proteins.^38^ Preclinical trials on WEL suggested an improvement in body weight may be due to reversing gluconeogenesis, glycogenolysis, and proteolysis.

Insulin plays a significant role in the metabolism of liver glycogen *via* the regulation of glycogenesis, which controls the above metabolism by standardizing the conversion of the dependent form of glucose-6-phosphate into the independent form of glucose-6-phosphate of glucose synthase.^39^ Glycogen synthetase enzyme increases glycogenesis, which plays a key role during the expansion of diabetes *via* converting glycogen into glucose, resulting also in boosting the blood glucose concentration. During diabetes, an increased number of glycosylation proteins is observed. Skeletal muscle maximum utilizes the glycogen as an express reflection of insulin activity, as insulin enhances intracellular glycogen deposition *via* a reduction of glycogen phosphorylase and by boosting glycogen synthase.^40,41^ Glycosylated hemoglobin is considered to be a significant marker of glycemic control. STZ-induced DM rats showed reduced liver glycogen activity, which was significantly (*p* < 0.001) enhanced by WEL treatment in a dose-dependent manner, indicating the reduced glucose synthetase enzymes during treatment. The possible mechanism of action may be due to a reduction in the absorption of glucose from the intestine or by a reduction of neoglucogenesis similar to with the standard drugs.

The liver plays a crucial role in the synthesis of glucose metabolism and postprandial hyperglycemia. In the liver, glucose is utilized to renovate the glucose into glucose-6-phosphatase with the help of hexokinase. The insufficiency of hexokinase results in a reduction of glycolysis and a significant downregulation of glucose utilization for energy production.^42,43^ Glucose-6-phosphatase converts fats into carbohydrates and during the type II DM augments the level of glucose-6-phosphatase, boosting the turning of fats into carbohydrates and starting deposition into the hepatic and renal tissue and inhibiting the action of hexokinase. Hexokinase, glucose-6-phosphatase and fructose-1-6-phosphate enzymes play key roles in the gluconeogenic pathway.^44,45^ Glucose-6-phosphatase and fructose-1-6-phosphate levels reach extended levels due to the boost in the synthesis of enzymes, which results in the upregulatation of glucose production *via* the liver during diabetes. STZ-induced type II DM group rats showed a reduced level of hexokinase and increased level of glucose-6-phosphatase and fructose-1-6-phosphate, which was dose-dependently altered by the WEL, with an almost similar result observed in the glibenclamide-treated group rats. The possible mechanism of action of this may be attributed to enhanced glycolysis and reduced gluconeogenesis.^44,45^ Glucose-6-phosphate dehydrogenase is considered as the first regulatory enzyme of the pentose phosphate pathway. STZ-induced type II DM group rats showed the reduction of glucose-6-phosphate dehydrogenase activity, possibly due to the reduction of hexose monophosphate shunt (HMP), therefore reducing the generation of equivalents, including NADH and NADPH, which are necessary for the glutathione reductase and thereby GSH activity in tissues. The reduced activity of NADPH in the cells makes them more susceptible to inducing oxidative damage^46^. STZ-induced type II DM group rats demonstrated a reduced activity of glucose-6-phosphate dehydrogenase, which was dose-dependently boosted by the WEL. The possible mechanism for this may be the increased production of hydrogen, which further binds to the NADP^+^ and produces the NADPH, which further induces the synthesis of fats from the carbohydrate and decreases the plasma glucose to a considerable extent. According to Ahmed *et al.*, the plasma insulin level is controlled by the reduced activity of glucose-6-phosphatase and fructose-1-6-phosphate, and the same results were found in our study, which suggests that WEL increased the plasma insulin level *via* the reduced activity of glucose-6-phosphatase and fructose-1-6-phosphate.

Mitochondrial dysfunctions have been linked to the progression of various types of pathologies, such as diabetes. Mitochondrial abnormalities may contribute to metabolic deformity and emanate insulin resistance and increase the chance of developing diabetes. Generally, mitochondrials generate the energy for cells and work as the powerhouse of the cell. In an inefficient endogenous antioxidant system, this occurs due to a low consumption of ATP production/oxygen, which leads to the production of superoxide anions.^47,48^ The generation of superoxide radicals increases the oxidative stress load and leads to the formation of reactive oxygen species (ROS). ROS formation may boost the production of pro-inflammatory cytokines and the rate of mutagenesis. Mitochondrial generation of ATP occurs less in cells, which further increases the amount of lipids and glucose in the cell. Previously published literature suggest that insulin resistance is connected to the reduced mitochondrial oxidative enzymes, mitochondrial number, and reduced ATP synthesis in human muscle. In the current experimental investigation, we showed the dual mechanism action as either an antioxidant or anti-inflammatory mechanism.^48–50^ The anti-inflammatory potential of WEL gives support to our hypothesis. Several previous published literature reports have suggested that the free radical/reactive oxygen species/reactive nitrogen species start the production of oxidative stress, which plays a significant role in the expansion of hyperglycemia and its complications. A variety of mechanisms are involved in the production of ROS during diabetes, including glucose auto-oxidation, lipid peroxidation (LPO), production of glycation products, and protein glycation.^50–52^ STZ induces toxicity in the pancreatic β-cells and starts the secretion of insulin and increases the overload of ROS/free radicals. Decreased levels of endogenous antioxidants then start the generation of superoxide (O_2_) and hydrogen peroxide (H_2_O_2_) and induce the tissue and cellular damage, causing the diverse diabetes conditions. SOD and CAT are considered as the significant primary endogenous antioxidant enzymes. Superoxide radicals damage the cellular membrane and biological structure of cells. SOD converts the superoxide into H_2_O_2_. Another endogenous antioxidant CAT switches H_2_O_2_ into hydroxyl radicals and converts this in tissue to highly reactive hydroxyl radicals.^22^ Polyunsaturated fatty acids play a significant role in the production of ROS, whereby ROS react with the cell membrane and all biological substances and induce lipid peroxidation. The upregulation of the LPO level impairs the membrane-bound enzyme activity *via* reducing the membrane fluidity. Researchers are now targeting the estimation of the MDA level (as an indicator of LPO) for estimation of an increased overload of LPO during type II DM.^1,2^ STZ-induced type II DM group rats showed an amplified level of MDA, which was significantly (*P* < 0.001) downregulated by the WEL, suggesting an anti-diabetic effect.

In addition to hyperglycemia, high levels of TC and TG can be found in the blood of type II DM due to a change of lifestyle and high intake of junk foods and fats. The lipid abnormality occurs in diabetic patients due to the insensitivity of the peripheral tissue of insulin.^20,53^ Insulin plays an imperative role in the downregulation of hormone-sensitive lipase enzyme and in the activation of lipoprotein lipase. Previous literature suggests that diminution of free cholesterol and free acids *via* pancreatic cholesterol esterase and pancreatic lipase downregulate the hyperlipidemia linked with the DM. STZ-induced type II DM group rats showed increased levels of total cholesterol and LDL, which was significantly (*p* < 0.001) reduced by the WEL in a dose-dependent manner. The possible mechanism of action may be due to the inhibition of pancreatic cholesterol esterase and pancreatic lipase. During the DM, the deposition of free fatty acids is increased in the peripheral tissue and boosts the level of triglycerides in the serum.

The inflammatory mediator and inflammation reaction play a crucial role in the development of diabetes and its complications. Inflammation reaction decreases insulin sensitivity and reduces the sensitivity of lipase tissue. In normal conditions, an inflammatory marker, such as TNF-α and CRP, can be found in a low amount in the circulation, leading to a low grade of inflammation vascular damage.^1^ Normally, CRP secreted from the hepatic tissue plays an important role in the secretion of TNF-α and IL-6. But several researchers suggested that an augmented level of CRP is directly linked with insulin resistance, obesity, glucose tolerance, BGL, and the etiology of type II DM; the same result was observed in the STZ-induced type II DM group, and WEL treatment significantly (*P* < 0.001) decreased the CRP level.^54,55^ The regeneration of pancreatic β-cells suggests the antidiabetic effect of WEL. During diabetes, an increased level of TNF-α showed the effect on the insulin receptor and decreased the insulin sensitivity, while IL-6 shows the effect on the glucose *via* altering the insulin receptor, IRS, glut-4, which increase the incidence of insulin resistance. In our study, we observed increased levels of TNF-α and IL-6, which suggests the resistance of the insulin level in the STZ-induced type II DM group, while the WEL treatment demonstrated the reduced level of an inflammatory mediator. The improved insulin secretion provides support for our hypothesis.

## Conclusion

The result of the current experimental study suggested the antihyperglycemic effect and *in vitro* inhibition of DPPIV suggesting the antidiabetic effect of WEL. Several previous studies showed that standard drugs, such as glibenclamide, have several side effects, such as headache, nausea, and low blood sugar level, when taken in a higher amount. Based on our results, we can conclude that WEL might be useful in the treatment and prevention of diabetes and its complications, such as insulin resistance and spoiled glucose tolerance with a DPP-IV inhibitory effect.

## Conflicts of interest

None, all authors declare none conflict of interest.

## Abbreviations

WEL: Wedelolactone
DPPIV: Dipeptidyl peptidase-4
GLUT1: Glucose transporter 1
PPARγ: Peroxisome proliferator-activated receptors-γ
OGTT: Oral glucose tolerance test
STZ: Streptozotocin
DM: Diabetes mellitus
CRP: C-reactive protein
TNF-α: Tumor necrosis factor alpha
IL-6: Interleukin 6
PTP1B: Protein tyrosine phosphatase 1B
BGL: Blood glucose level
iPH: Ponderal homogeneity index
PG: Ponderal grain
AST: Alkaline phosphatase
AST: Aspartate aminotransferase
ALT: Alanine transaminase
BUN: Blood urea nitrogen
TP: Total protein
TC: Total cholesterol
HDL: High density lipoprotein
TG: Triglyceride
VLDL: Very low density lipoprotein,
LDL: Low density lipoprotein
MDA: Malonaldehyde
GPx: Glutathione peroxidase
SOD: Superoxide dismutase
CAT: Catalase

## Supplementary Material

RA-008-C7RA12568B-s001
