# Supplementary material for: Deconvoluting the dual hypoglycemic effect of wedelolactone isolated from Wedelia calendulacea: investigation via experimental validation and molecular docking
Source: RSC Adv. 2018 May 18;8(32):18180–96. doi: 10.1039/c7ra12568b (PMC9080591; doi:10.1039/c7ra12568b)
Supplement: RA-008-C7RA12568B-s001 [file RA-008-C7RA12568B-s001.pdf]

Electronic Supplementary Material (ESI) for RSC Advances.
This journal is © The Royal Society of Chemistry 2018

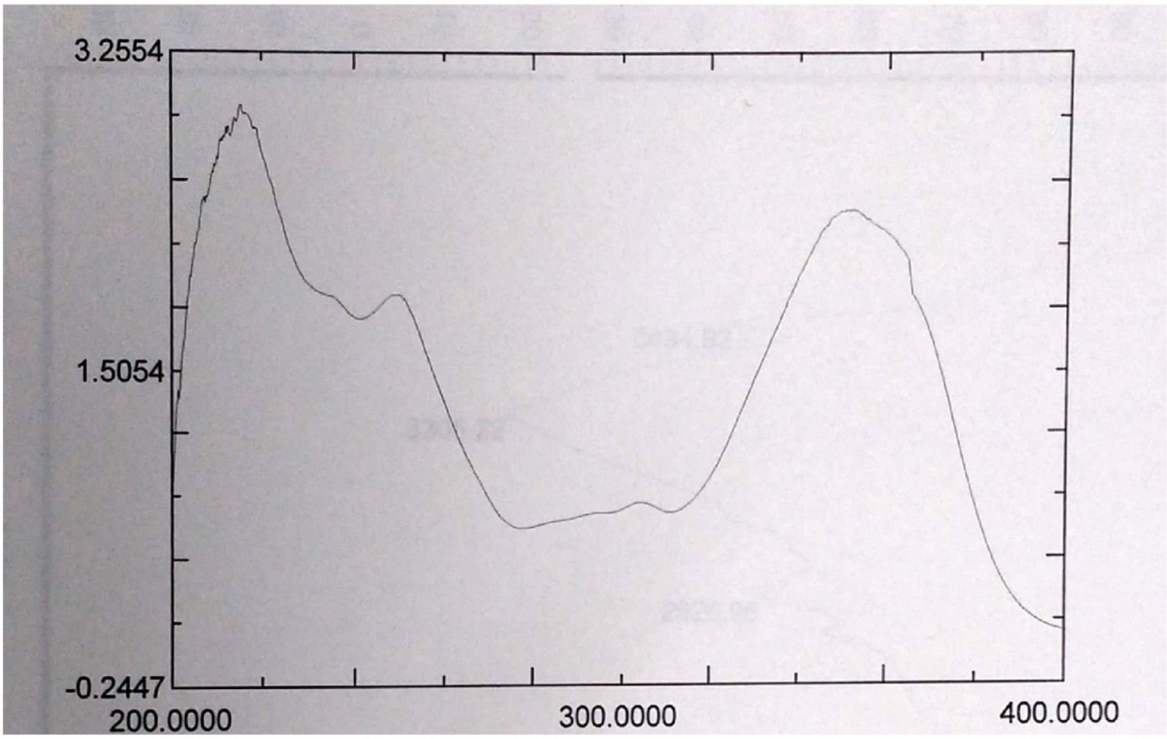

Supplementary figure 1: UV spectra of compound

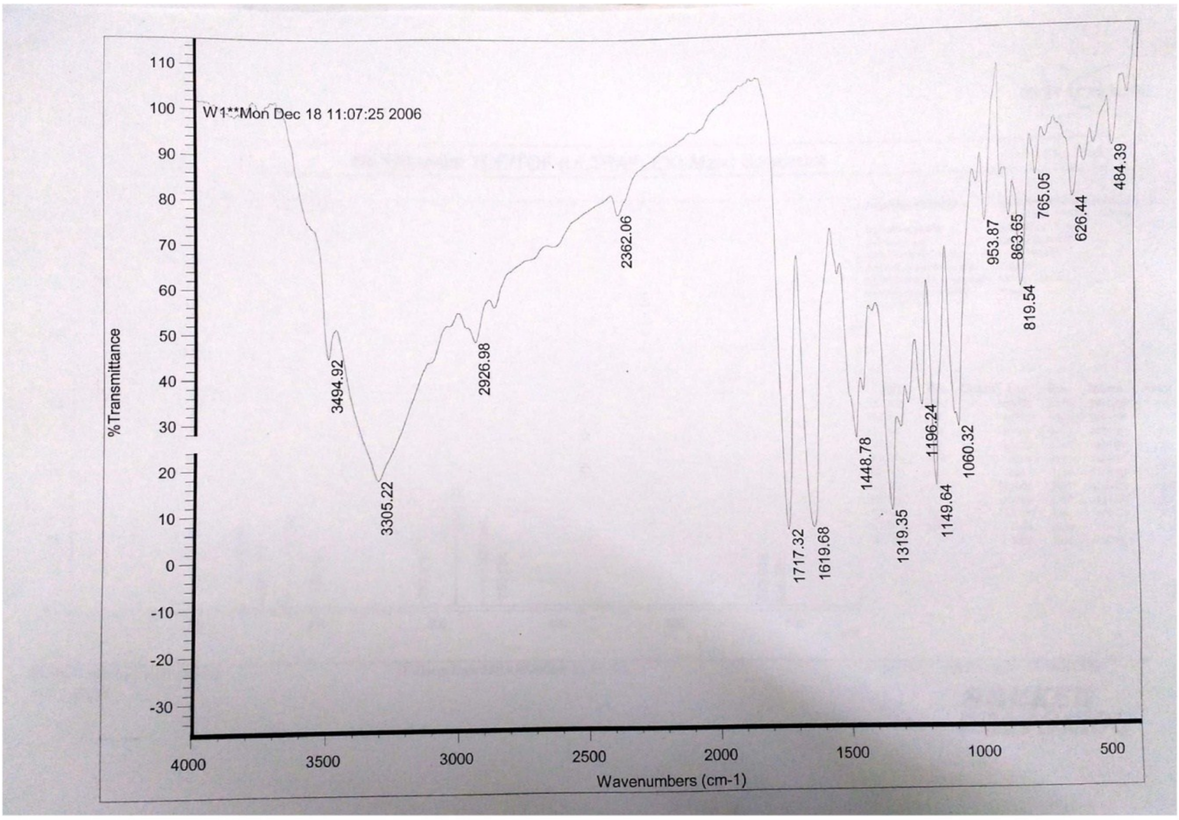

Supplementary figure 2: FTIR spectra of compound

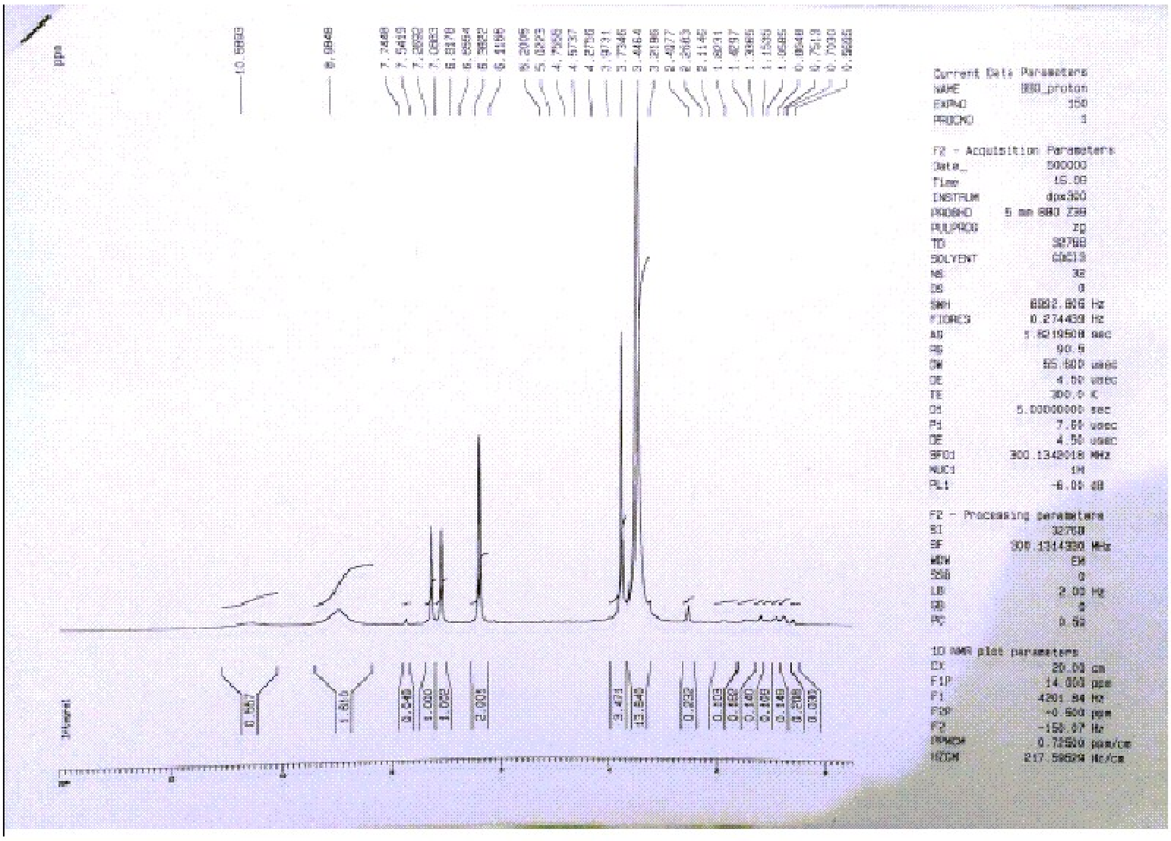

Supplementary figure 3: NMR spectra of compound

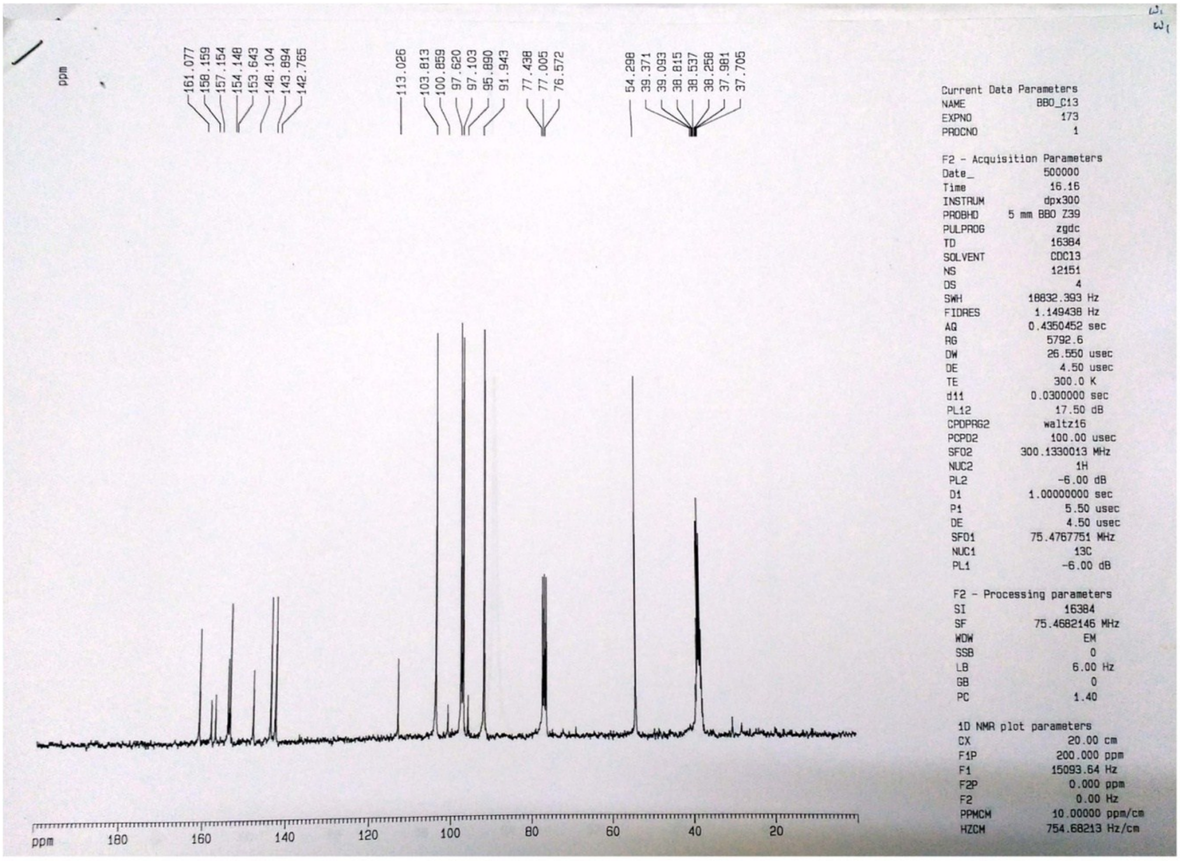

Supplementary figure 4: NMR spectra of compound

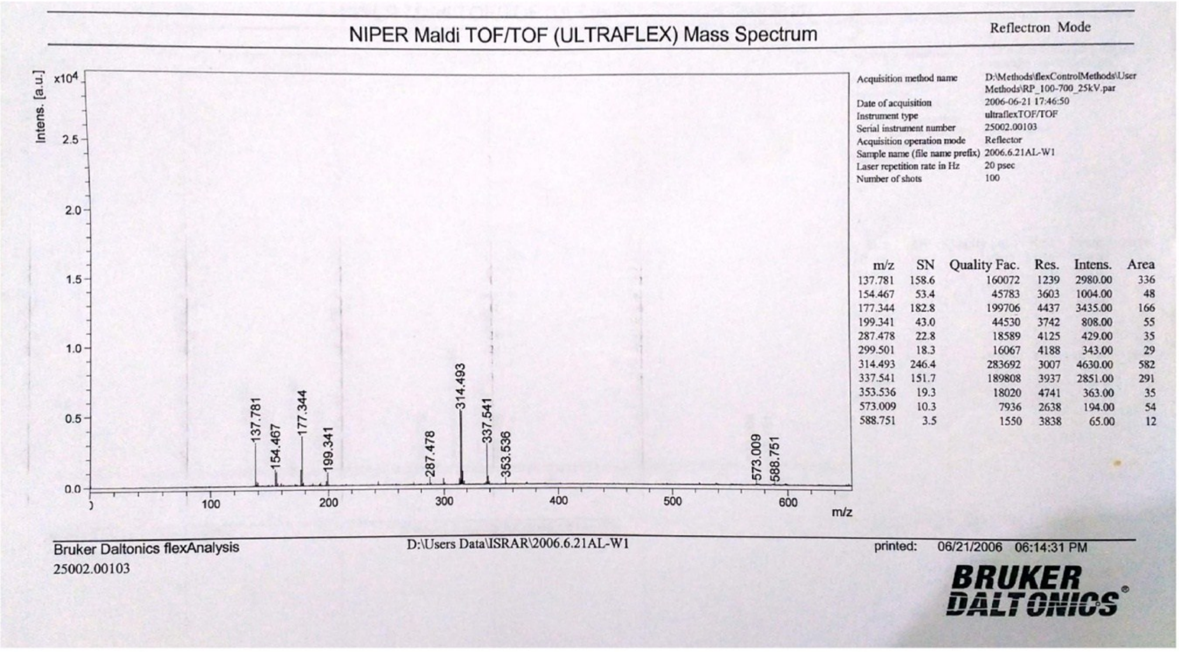

Supplementary figure 5: Mass spectra of compound

Supplementary table 1: 1D NMR spectral data of Compound WC-1.

Position	^1H NMR	^1H NMR[56]	^{13}C- NMR	^{13}C- NMR[56]
1	-		-	
2	-		161	161.92
3	-		154	154.45
4	-		100.8	101.38
5	-		153	154.45
6	6.38 (*d*, *J* = 2.0)	6.62 *s*	97.1	97.84
7	-		158	158.59
8	6.38 (*d*, *J* = 2.0)	7.24 *s*	97.6	98.57
9	-		95.8	96.40
10	7.08 *s*	-	91.9	92.94
11	-	-	143.8	148.55
12	-	-	142.7	145.10
13	7.26 *s*	6.46 *s*	103	104.24
14	-	-	157	157.45
-	3.73 *s* at OCH_3	9.82 s at OCH_3	54.2	55.43
-	8.98 *brs* at 11, 12 (OH)	9.34 *s*, 9.36 *s* at 11, 12 (OH)	-	-
-	10.58 *s* at 5 (OH)	10.97 s at 5 (OH)	-	-
